# Symptom clusters and survival in Portuguese patients with advanced cancer

**DOI:** 10.1002/cam4.860

**Published:** 2016-09-13

**Authors:** Pedro C. Barata, Alice Cardoso, Maria P. Custodio, Marta Alves, Ana L. Papoila, Barbosa António, Peter G. Lawlor

**Affiliations:** ^1^ Medical OncologyCentro Hospitalar Lisboa CentralLisbonPortugal; ^2^ Palliative CareCentro Hospitalar Lisboa CentralLisbonPortugal; ^3^ Epidemiology and Statistics UnitCentro Hospitalar Lisboa CentralLisbonPortugal; ^4^ Centre of BioethicsFaculty of MedicineLisbon UniversityLisbonPortugal; ^5^ Bruyere Continuing CareDivision of Palliative CareDepartment of MedicineBruyere and Ottawa Hospital Research InstitutesUniversity of OttawaOttawaCanada

**Keywords:** Advanced cancers, palliative care, solid tumors, survival, symptom clusters

## Abstract

This study aimed to identify clusters of symptoms, to determine the patient characteristics associated with identified, and determine their strength of association with survival in patients with advanced cancer (ACPs). Consecutively eligible ACPs not receiving cancer‐specific treatment, and referred to a Tertiary Palliative Care Clinic, were enrolled in a prospective cohort study. At first consultation, patients rated 9 symptoms through the Edmonton Symptom Assessment System (0–10 scale) and 10 others using a Likert scale (1–5). Principal component analysis was used in an exploratory factor analysis to identify. Of 318 ACPs, 301 met eligibility criteria with a median (range) age of 69 (37–94) years. Three SCs were identified: neuro‐psycho‐metabolic (NPM) (tiredness, lack of appetite, lack of well−being, dyspnea, depression, and anxiety); gastrointestinal (nausea, vomiting, constipation, hiccups, and dry mouth) and sleep impairment (insomnia and sleep disturbance). Exploratory factor analysis accounted for 40% of variance of observed variables in all SCs. Shorter survival was observed for patients with the NPM cluster (58 vs. 23, *P* < 0.001), as well as for patients with two or more SCs (45 vs. 21, *P* = 0.005). In a multivariable model for survival at 30‐days, age (HR: 0.98; 95% CI: 0.97–0.99; *P* = 0.008), hospitalization at inclusion (HR: 2.27; 95% CI: 1.47–3.51; *P* < 0.001), poorer performance status (HR: 1.90, 95% CI: 1.24–2.89; *P* = 0.003), and NPM (HR: 1.64; 95% CI: 1.17–2.31; *P* = 0.005), were associated with worse survival. Three clinically meaningful SC in patients with advanced cancer were identifiable. The NPM cluster and the presence of two or more SCs, had prognostic value in relation to survival.

## Introduction

Cancer patients experience many concurrent symptoms that significantly compromise their emotional and functional status and their quality of life 1, 2. As patients rarely present with a single symptom, there is a perceived need to shift the paradigm of symptom management research from trying to understand any one particular symptom in isolation to a broader focus on evaluating the relationship among multiple symptoms 1.

As a dynamic construct, the term symptom cluster (SC) has been defined as two or more interrelated symptoms that present together, independent of other SCs, and may possibly suggest a common etiology or underlying mechanism 3. It is possible to identify SCs in both cancer and non‐cancer patients 4. In oncology, the investigation of SCs has been performed mostly on early‐stage cancer 5, 6, specific primaries 7, 8, and specific metastatic sites 9, 10. However, there is a paucity of such studies in advanced cancer patients (ACPs) who are currently neither receiving chemo‐ nor radiotherapy and have high symptom burden 11, 12.

Overall survival is an important endpoint for patients with advanced cancer. Recent studies have shown that adequate symptom management of ACPs offers better quality of life and improves prognosis 13. In advanced cancer, individual symptom burden in different cancers has been associated with poor survival 14, 15. Despite some studies suggesting that SCs are associated with poor survival in patients with specific primary cancers, the real impact of clusters on survival in cancer in general is still lacking 16. A better knowledge of SC is crucial in the development of novel treatments in symptom management, which results in a significant benefit for patients with advanced cancer.

This study aimed to (1) identify the presence and composition of SCs in ACPs who were not receiving any type of anticancer treatment; (2) determine the patient characteristics associated with identified SCs; and (3) examine the strength of association of identified clusters with survival.

## Methods

### Study setting

The Hospital Santo António dos Capuchos (HSAC) is part of Central Lisbon Hospital Center, located in Lisbon, Portugal. The palliative care program at HSAC includes a consultation service that assesses urgent cases on a same‐day basis, and a daily weekday palliative care outpatient clinic. Hospitalized patients with cancer were also referred to the palliative care service by other specialties. Consecutive referrals from among those admitted to HSAC and those referred by oncologists to the palliative care outpatient clinic were screened for study eligibility.

### Subjects and eligibility

We conducted a prospective cohort study of consecutive ACPs who were referred to our palliative care program between October 2012 and May 2015 at the Department of Medical Oncology, HSAC. In this hospital, ACPs are commonly referred to Palliative Care Department for symptom management or for transition from the hospital to home or hospice care. These referrals may happen at different times in the disease trajectory, according to physician′s criteria. To meet this study, patients were: (1) aged 18 years or older; (2) evidence on diagnostic imaging of progressive advanced solid cancer with loco‐regional or distant metastatic disease; (3) absence of anticancer therapy at time of referral; (4) no evidence of dementia or delirium on assessment with the Portuguese versions of the Short Portable Mental Status Questionnaire (SPMSQ) 17 and the Confusion Assessment Method (CAM) 18, respectively; and (5) ability to provide verbal or written answers to assessment measures and to sign written consent. Patients with hematological malignancies were excluded. All patients were enrolled and assessed at initial consultation with the palliative care service. This study was part of a longitudinal project that was approved by the hospital research ethics committee and informed consent of patients was obtained. This study reports on baseline cross‐sectional data assessed at the initial consultation and survival.

### Study measures and data collection

Baseline patient demographics were collected at initial palliative care consultation. Data on patients′ primary cancer location, metastatic sites and number, and type and date of last active cancer‐specific treatment (chemotherapy, radiotherapy or other cancer‐specific therapies such as tyrosine kinase inhibitors or endocrine therapies) were also gathered. Functional performance status (PS) was assessed with the Eastern Cooperative Oncology Group (ECOG) scale 19. Cognitive status was evaluated with the Portuguese version of SPMSQ 17. The validated Portuguese version of the CAM was used to screen for delirium 18. Subjects meeting the study eligibility criteria had a comprehensive assessment of their symptom profile.

A total of 19 symptoms were assessed. All patients assessed by the palliative care team were requested to complete the revised version of the Edmonton Symptom Assessment Scale (ESAS) and 10 other symptoms using a Likert scale (1–5), as a routine component of the initial and subsequent consultations. The ESAS is a standardized 0–10 numerical rating tool (0 = not a problem, 10 = worst imaginable level of symptom) that is commonly used by palliative care teams to evaluate the intensity of nine symptoms: pain, dyspnea, lack of appetite, nausea, fatigue, drowsiness, anxiety, depression, and general well‐being 20, 21. It has been revised to facilitate its use (ESAS‐r) 22, and this version of the instrument, used in this study, has been translated into Portuguese 23. The ESAS‐r was supplemented by questions about the following symptoms: dry mouth, vomiting, constipation, hiccups, sweating, weight loss, dysphagia, sleep disturbance, insomnia, and lack of memory. The intensity of these 10 symptoms was scored on a Likert scale: not at all = 1, a little = 2, moderate = 3, severe = 4, and extremely stressing = 5. Scores rated >2 on ESAS‐r or >1 on Likert scale were deemed to be clinically significant and designated as measures of prevalence.

Our electronic records system was used to obtain laboratory data and to complete clinical data collected with patient forms.

### Statistical analysis

Descriptive statistics were used to summarize baseline patient demographics, disease characteristics, and distributions of ESAS‐r and Likert scores. The scores from both scales were normalized using z scores. Categorical data were analyzed using the Chi‐Square test. A principal component factor analysis with varimax rotation was conducted on the standardized clinically significant intensity scores of the 19 symptoms to identify the SCs. The Kaiser‐Meyer‐Oklin measure was calculated to assess sampling adequacy (scores > 0.60 indicate adequate sample size for the analysis). A factor loading > 0.40 was considered as an inclusion criterion for each symptom. Derived factors were interpreted and discussed among the research team, and final factor structure included factors deemed to be clinically as well as statistically relevant. The internal consistency and reliability of the derived SC were assessed with the Cronbach's alpha coefficient.

Survival analysis was based on the time elapsed between the date of study inclusion and the date of death or last follow‐up. Kaplan–Meier estimator was applied to obtain group survival estimates and the log‐rank test was used to compare survival between groups. Univariable and multivariable Cox regression models were fitted to the data considering time to death at 30 days. Among those variables that could influence the time to death at 30 days, a new variable (for each SC), characterizing each patient as having or not having a SC, was considered. Arbitrarily, the presence of at least 75% of the symptoms composing each SC was a set requirement for designating the presence of that given SC; otherwise, it was considered to be absent. The level of statistical significance for analyses was set at *α *= 0.05. The statistical analysis was performed using SPSS version 21.0 (SPSS Inc., Chicago, IL).

## Results

**Figure 1:**
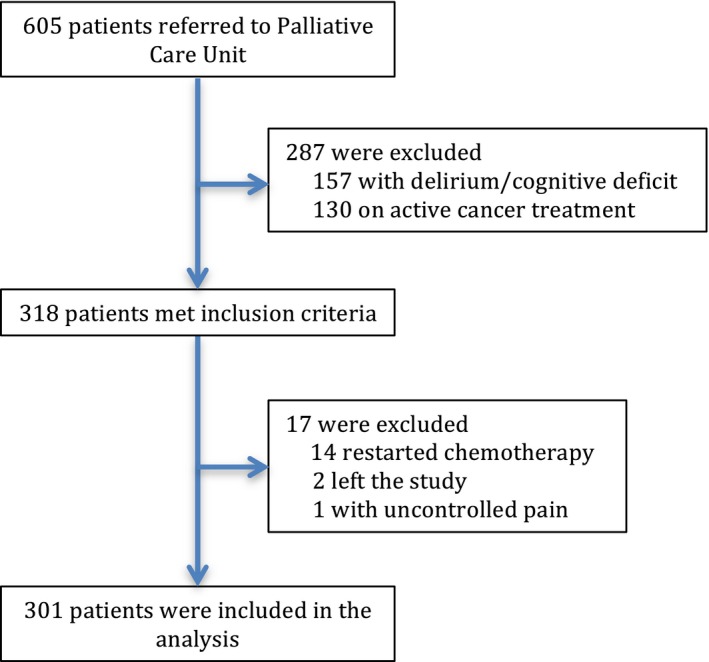
Study participant flow diagram.

Of the 605 patients referred to the palliative care program, 157 were excluded due to cognitive deficit or delirium and 130 were on active cancer treatment (Fig. 1). Our analyses were conducted on the initial symptom assessments from 318 patients with advanced solid tumors. Among these, a further 17 were excluded: 14 restarted cancer treatment, 2 left the study, and 1 did not complete the questionnaire due to severe pain. Three hundred and one patients were included in the final analysis.

The baseline demographic details of our study sample are summarized in Table 1. The median age was 69 years (range: 37–94 years) and 172 (57.1%) were men. The most common primary cancer sites were gastrointestinal (27.6%), lung (17.6%), breast (15.6%), and genitourinary (13.0%). The majority of patients (67.4%) had two or more metastatic sites. Patients with no metastases included those with locally advanced disease and no disease elsewhere. A total of 65% of patients had a PS of 3 or 4.

**Table 1:** Baseline patient characteristics

Clinical characteristics	Number (%) of patients
Gender	
Male	172 (57.1)
Female	129 (42.9)
Age	
Median (years)	69
Range	37–94
ECOG	
0	3 (1.0)
1	15 (5.0)
2	88 (29.2)
3	142 (47.2)
4	53 (17.6)
Hospital status	
Outpatient	106 (35.2)
Inpatient	195 (64.8)
Primary cancer site	
Biliary tract	12 (4.0)
Breast	47 (15.6)
Gastrointestinal	83 (27.6)
Genitourinary	39 (13.0)
Gynecological	13 (4.3)
Hepatocarcinoma	14 (4.7)
Lung	53 (17.6)
Pancreas	21 (7.0)
Unknown	9 (3.0)
Other^a^	10 (3.3)
Number of metastatic sites	
0	13 (4.3)
1	37 (12.3)
>1	251 (83.3)

a This group includes central nervous system, head and neck, skin mesothelioma and sarcomas. ECOG, Eastern Cooperative Oncology Group.

In Table 2, symptom prevalence and severity are described. The median number of symptoms was 9 (0–18) and the most common symptoms were tiredness, pain, somnolence, dry mouth, and weight loss, ranging in prevalence from 80.7% to 100%.

**Table 2:** Symptom clusters composition and relative frequencies

Symptom	Symptom prevalence (%)	Mean score (SD)	Median score (P_25_–P_75_)
ESAS			
Pain	251 (83.3)	5.8 (2.2)	6 (4–8)
Tiredness	301 (100)	6.2 (2.9)	7 (4–9)
Somnolence	249 (82.7)	4.6 (3.2)	5 (2–7)
Nausea	91 (30.2)	5.9 (2.2)	7 (4–8)
Lack of appetite	225 (74.8)	7.0 (2.2)	7 (5–9)
Dyspnea	91 (30.2)	7.0 (2.3)	7 (5–8)
Depression	225 (74.8)	7.0 (2.1)	7 (5–8)
Anxiety	178 (59.1)	6.3 (2.2)	7 (4–8)
Lack of well‐being	234 (77.8)	6.3 (2.0)	7 (5–8)
Likert scale			
Vomiting	75 (24.9)	2.8 (0.9)	3 (2–3)
Constipation	160 (53.2)	3.2 (1.0)	3 (2–4)
Weight loss	243 (80.7)	3.5 (0.9)	4 (3–4)
Dysphagia	89 (29.6)	3.1 (1.1)	3 (2–4)
Dry mouth	249 (82.7)	3.4 (1.0)	4 (2–4)
Sweating	63 (20.9)	2.8 (1.0)	3 (2–3)
Hiccups	63 (20.9)	2.8 (1.0)	3 (2–3)
Insomnia	173 (57.5)	3.1 (0.9)	3 (3–4)
Sleep disturbance	159 (52.8)	3.2 (0.9)	3 (3–4)
Lack of memory	115 (38.2)	2.7 (0.9)	2 (2–3)

Symptom is considered prevalent if ESAS score > 2 or Likert score > 1.

ESAS, Edmonton Symptom Assessment Scale; SD, Standard deviation.

Table 3 shows the three identified SCs that accounted for 39.7% of the total variance in symptom scores. The neuro‐psycho‐metabolic (NPM) cluster included tiredness, lack of appetite, dyspnea, anxiety, and lack of well‐being; the gastrointestinal (GI) cluster included nausea, vomiting, constipation, dry mouth, and hiccups; and sleep impairment (SI) cluster included insomnia and sleep disturbance. The NPM, GI and SI clusters were present in 131 (43.5%), 51 (16.9%), and 139 patients (46.2%), respectively. One hundred and five patients (34.9%) had one SC, 79 (26.2%) had two, and 18 (6.3%) had all SCs. The NPM, GI, and SI clusters demonstrated internal consistency and validity with good Cronbach′s alpha coefficient values of 0.74, 0.59, and 0.86, respectively. The same cluster analysis to the subgroup of patients with GI cancers (*n* = 133) was performed, and the same three clusters were identified (Cronbach′s alpha of 0.76, 0.67 and 0.85, respectively), although this subgroup differed by having more men (65% vs. 57%), better PS (43% vs. 35% with ECOG 0–2), and more with an outpatient designation (42% vs. 35%) when compared to the general study population, respectively.

**Table 3:** Frequency of the different symptom clusters identified

Symptoms	N (%) of patients	Factor loading score	% of variance	Cronbach's *α*
Neuro‐psycho‐metabolic cluster	131 (43.5)		17.31	0.736
Tiredness		0.718		
Lack of appetite		0.498		
Dyspnea		0.538		
Depression		0.684		
Anxiety		0.618		
Lack of well‐being		0.703		
Gastro‐intestinal cluster	51 (16.9)		12.39	0.591
Nausea		0.600		
Vomiting		0.758		
Constipation		0.522		
Dry mouth		0.410		
Hiccups		0.545		
Sleep impairment cluster	139 (46.2)		9.99	0.864
Insomnia		0.869		
Sleep disturbance		0.828		

The frequency of SCs among different primary tumors was analyzed in cancers with a study sample prevalence of 1%: lung, gastrointestinal, breast, and genitourinary tumors. The cluster NPM was present in 57% of lung and breast cancers, and in 37% and 31% of GI and GU cancers, respectively. The GI cluster was rarely present in lung cancer (4%) and less frequent in breast and GU cancers (13%) than reported for all cancers. The highest NPM frequency was observed in GI cancers (27%). The frequency of the SI cluster was very similar in all groups; its presence ranged between 41% and 49% of the cases.

We performed an exploratory analysis to assess the factors related to the presence of one SC in particular. This analysis included tumors with at least 45 patients: lung, breast, and gastrointestinal cancers. The cluster NPM was more common (76% vs. 24%, *P* = 0.028) in patients with PS 3 and 4 versus 0–2, and in those hospitalized versus not hospitalized (75% vs. 25%, *P* = 0.031). Patients with gastrointestinal cancer had the NPM cluster and GI cluster less frequently, with presence versus absence of 37% versus 63% and 27% versus 73%, respectively (*P* < 0.001).

**Figure 2:**
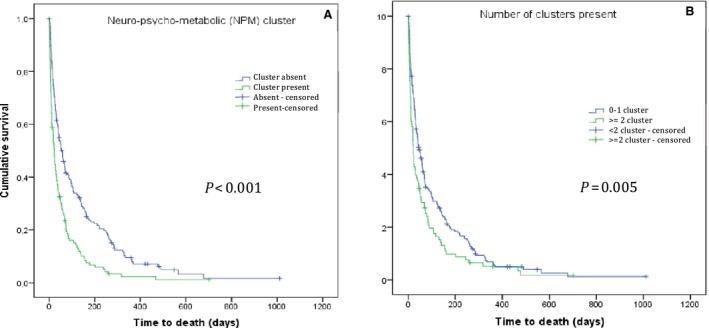
Kaplan–Meier survival estimates according to the presence of symptom cluster. (A) Neuro‐psycho‐metabolic (NPM) cluster. (B) Number of symptom clusters present.

Finally, the strength of association of the SCs with survival was analyzed. The median survival of the whole cohort was 37 (95% CI: 28–46) days. A statistically significant reduction in survival was observed for patients with the NPM cluster compared to those without the cluster (58 days vs. 23 days, *P* < 0.001, see Fig. 2A). No difference in survival was observed for the presence of the GI and SI clusters, nor the cancer specific‐treatment received (*P* = 0.788) before inclusion. The number of SCs present in any given patient was related to survival: patients with two or more clusters lived a median of 21 (95% CI: 17–25) days, compared with a median of 45 (95% CI: 32–58) days for those with one or zero clusters (*P* = 0.005) (Fig. 2B).

In the univariable Cox regression analysis, variables potentially influencing time to death at 30 days, with a *P* < 0.25, were selected for the multivariable model: age, PS, hospital status, and NPM and SI clusters. Remarkably, the variables sex, site of metastases, GI cluster, and cancer‐specific treatment were not significantly associated with time to death. In the multivariable model, younger age (HR: 0.98; 95% CI: 0.97–0.99; *P* = 0.008), hospitalization at inclusion (HR: 2.27; 95% CI: 1.47–3.51; *P* < 0.001), poorer PS (HR: 1.90, 95% CI: 1.24–2.89; *P* = 0.003), and NPM cluster presence (HR: 1.64; 95% CI: 1.17–2.31; *P* = 0.005), were independently associated with worse survival at 30 days.

## Discussion

The study of SCs may be particularly relevant for patients with advanced cancer because they frequently report multiple, concurrent symptoms that are difficult to control 24. As in previous reports, people with advanced cancers are polysymptomatic, a common feature across all primary sites 25, 26.

We investigated SCs in a sample of patients with advanced cancer, different solid tumors, and not receiving any type of disease‐modifying, anti‐cancer treatment.

Among all of the 19 symptoms that were assessed, six (pain, somnolence, weight loss, dysphagia, sweating, and lack of memory) were not included in any of the clusters identified in our study. Although these exclusions were not particularly surprising for most of these symptoms, we would have expected pain—to be identified as part of a cluster or associated with other clusters identified, as reported in previous studies 11, 27. The lack of association of pain with depression, anxiety, or lack of well‐being was also noted in a recently published Portuguese study of cancer pain 28; the authors questioned the possibility of cultural explanations or cultural misinterpretation in the context of some assessment tools. Alternatively, our finding may suggest that the presumed strong relationship between those pain and psychological distress variables may, as suggested in previous studies, be somewhat exaggerated 11, 29. Meanwhile, it is possible that somnolence and weight loss may be viewed and rated with less and account for their failure to be included in a cluster, albeit that the characteristics of this heterogeneous population limit definitive conclusions on this regard.

The most common cluster, SI was present in almost half (46%) of our patients. The symptoms of insomnia and sleep disturbance that comprise this cluster are among some of the most frequent symptoms experienced by patients with cancer 30. Despite the paucity of studies on sleep disturbance in advanced cancer patients, a prospective study that assessed the prevalence of sleep disorder in an acute palliative care unit reported that 30% of patients slept <5 h 31. These data together with our findings support the need to incorporate attention to sleep disturbance into routine practice in supportive care.

The second most common cluster in our study, NPM (44%), has also been found in several other studies, in which its presence was either partially or totally represented 11, 12, 24, 32, 33, 34. Among four of these studies, which were conducted in patients with advanced cancer, one of them identified a cluster with 4 of the NPM symptoms that were identified in our current study;^33^ another identified 2 of our study's NPM symptom cluster^34^; the other two studies grouped the symptoms of depression and anxiety with sleep problems to comprise a distinct symptom cluster in their studies 11, 12. The combination anorexia‐tiredness is also described as an anorexia‐cachexia cluster in other studies 11, 12. This anorexia‐tiredness syndromal combination may be associated with among other factors, cytokine release, such as C‐RP, interleukin‐6, and tumor necrosis factor‐alpha 34, 35. Meanwhile, given previously published data, the association of anorexia‐cachexia with anxiety and depression was not a surprise. In a large prevalence study of advanced cancer patients with different cancers, the factors poor PS, anorexia, and anxiety were independent predictors of depression, fatigue, and pain in multivariate analysis 35. However, the exact mechanism for this association is unknown and whether the psychological component is a cause or consequence of physical deterioration is not completely known.

Interestingly, among the published studies in patients with advanced cancer that assessed sleep‐related symptoms, those symptoms comprising the SI cluster in our study were integrated in NPM cluster 5, 12. Furthermore, the association between sleep issues and NPM was suggested in another study performed in advanced cancer patients admitted to a palliative care unit for pain management; awaking early, anxiety, and depression were among the factors significantly associated with less hours slept 31.

The GI cluster was present in 17% of our patients. While this cluster had the Cronbach′s alpha (0.59), the presence of this cluster in the subgroup of patients with GI malignancies was also statistically significant.

Furthermore, the GI cluster has already been reported in several studies among different populations worldwide, including the English, Chinese, and Filipino validation studies of the symptom assessment instrument from MD Anderson Cancer Center (MDASI) 36, 37, 38. In these studies, the GI cluster was observed in higher prevalence (ranging from 23 to 28%) than in our patients 11, 12, 32, 36, 37, 38. To the best of our knowledge, we are unaware of specific symptom clusters research on patients with GI‐advanced cancers; however, in many studies, GI malignancies are the most common primaries included 12, 24, 27.

It is noteworthy that the symptom constipation clustered together with the other gastrointestinal symptoms of the GI cluster. This finding suggests that “upper” and “lower” gastrointestinal symptoms may be inter‐related, which may have therapeutic implications. Even if they do not share the exact same pathophysiology, effective therapy may relieve one symptom and either relieve or prevent another, for example relief of constipation, thus preventing nausea caused by intestinal stasis or occlusion 11, 26. In most of the studies reporting a GI cluster, mean scores for nausea and vomiting were significantly higher in advanced cancer patients receiving chemotherapy 11, 39, 40. Furthermore, in the study where chemotherapy was not a factor influencing the GI cluster, there was a significant proportion of patients with PS 0 or 1, and the majority of them had early‐stage disease 36. The exclusion of patients on cancer‐specific treatments as a criterion to be eligible for participation in our study may possibly explain the lower prevalence of the GI cluster that we observed.

Among the most frequent cancers, we found that PS and hospital status were associated with specific SC. The NPM cluster occurred predominantly in hospitalized patients with poorer PS and with breast or lung cancer. These findings suggest that the NPM cluster may be associated with deterioration in physical and psychological functioning in patients with advanced cancer, especially in those with a thoracic neoplasm. Indeed, the association of this cluster with worse survival supports this finding. The GI cluster was more commonly observed in gastrointestinal cancers, which is unsurprising and in line with published data, showing a clear relationship between GI symptoms and the anatomic site of primary cancer 11, 12. We must acknowledge that many other factors with the potential to influence the clustering of symptoms have been identified in other studies, including age and gender; and other factors that were not formally evaluated in our study, such as psychological distress or fluid accumulation 24, 41.

In our study, short‐term survival was independently associated with younger age, poorer PS, and hospitalization. Contrary to what one might expect, younger patients had worse survival. These patients were more tired (*P* < 0.001) and depressed (*P* = 0.018) (data not shown), although no differences in NPM and age were identified. We admit that differences in primary site distribution may help to explain these findings, as liver and intrahepatic bile duct, breast, and upper GI malignancies, which affect more people younger than 70 years old, were more prevalent in our cohort than in general population 42. In one study by Walsh et al. 25, age was also identified as an independent predictor of morbidity, regardless of primary tumor location. Furthermore, the inclusion of age and PS in future studies addressing symptom management is suggested.

The presence of more than one cluster, as well as the NPM cluster had a negative association with survival. In fact, the association of symptom burden with survival has already been reported in a number of studies 41, 43, 44. However, very few studies investigated the prognostic importance of SCs in advanced cancer patients 12, 27. Our results support the clinical relevance of examining survival as an outcome of symptom cluster research. The prognostic value of SCs could be used to develop survival models, and thus provide useful information to guide both oncologists and palliative care experts in their clinical decisions.

The findings of this study are somewhat preliminary given the relative paucity of reported studies on SCs in advanced cancer and the lack of methodological consistency among reported studies. Indeed, different methods may identify different sets of clusters, and results may vary depending on the statistical analysis technique used 32, 45. We used principal component analysis, as it is one of the most common methods used in SC research. Because the analysis is based on the symptoms and scaling used in assessment tools, we admit that our 19‐item questionnaire may produce results different from those originating with a smaller or larger item symptom inventory, as already noted by different authors 32.

Certain characteristics of our study sample need to be acknowledged. Given the differences in pathophysiology, behavior, and clinical outcomes between hematological and solid tumors, we excluded patients with hematological malignancies in order to minimize potential differences in the study population. Furthermore, almost two‐thirds of the patients were hospitalized at time of inclusion, likely reflecting various complications associated with advanced cancer. However, all of them were admitted at the HSAC site, excluding those admitted at other hospitals from under the broader CHLC organization. This could lead to a selection bias of patients included in the study. Furthermore, CHLC is a recognized cancer center for GI malignancies, especially biliary tract, hepatic and pancreatic cancers, as well as thoracic malignancies. We admit that the proportion of different primary cancers seen in this study, such as the high proportion of liver/intrahepatic bile duct and pancreatic tumors, is not representative of the cancer population at large or comparable to other series, and thus it may have influenced the results. Despite these limitations, other investigators, in similar settings, have also described similar SCs. Furthermore, we have identified the same clusters in a more homogeneous group of patients with GI tumors, which supports the validity of these associations of symptoms. Nonetheless, these findings will need to be replicated in further studies to draw conclusions 11, 12, 32. Future studies stratifying patients according to clusters and see its impact on survival should be conducted to develop treatment strategies with a positive impact on outcomes. Finally, the prospective evaluation of SC over time has been rarely performed. To answer the question whether the prevalence and intensity of the clusters change overtime with disease evolution, this study is part of a longitudinal project with repeated measures over time.

These data have important implications for medical practice and clinical research. SCs may have prognostic value. The importance of regular assessment of symptom burden and SCs in clinical practice should not be underestimated. As recommended by other authors, the identification of clinically meaningful subgroups of patients that are in need of strategically targeted therapeutic intervention supports the need both for more rigorous exploratory studies and the inclusion of cluster assessment data in future treatment evaluation strategies.

## Conclusions

We identified three clinically meaningful SCs in patients with advanced cancer. The NPM cluster, the presence of two or more clusters, as well as age, performance status and hospitalization, had prognostic value. Further studies are needed to better characterize these clusters, their evolution over time, and evaluate potential therapeutic interventions.

## Conflicts of Interest

None declared.
